# Reversed sex disparities in heart failure mortality in Spain, 2000–2023: a population-based analysis of older adults

**DOI:** 10.3389/fpubh.2025.1645351

**Published:** 2025-09-01

**Authors:** Ángela Hurtado-Prieto, Eloísa Mariscal-López, Mario Gutierrez-Bedmar, Rosa López-Gigosos

**Affiliations:** ^1^Department of Public Health and Psychiatry, Faculty of Medicine, Universidad de Málaga, Málaga, Spain; ^2^Cardiology Service, Costa del Sol University Hospital, Marbella/Málaga, Spain; ^3^Biomedical Research Institute of Málaga and Nanomedicine Platform (IBIMA-BIONAND), Málaga, Spain; ^4^Centro de Investigación Biomédica en Red de Enfermedades Cardiovasculares (CIBERCV), Instituto de Salud Carlos III, Madrid, Spain

**Keywords:** heart failure, sex differences, mortality trends, health outcomes, aging adults

## Abstract

**Background and objectives:**

Heart failure (HF) is a growing public health concern in Spain, ranking as the third leading cause of cardiovascular mortality and contributing substantially to hospitalizations and outpatient visits. Clinical presentation, pathophysiology, therapeutic response, and healthcare access differ significantly between sexes. This study analyzes trends in HF mortality in Spain from 2000 to 2023, with a specific focus on sex-based disparities.

**Methods:**

We conducted a population-based descriptive analysis of annual HF mortality among individuals aged 50 and older in Spain, disaggregated by sex and five-year age groups. Mortality trends were examined over two periods (2000–2008 and 2009–2023) to assess patterns of change over time.

**Results:**

Overall, HF mortality has declined in Spain, with a sharper reduction from 2000 to 2008, followed by a more gradual decrease in subsequent years. Women exhibited a 34.4% higher average HF mortality rate compared to men, although this gap narrowed significantly over the study period. Age-stratified analysis revealed higher HF mortality in men aged 50–79, while in those aged 80 and older, women exhibited higher mortality.

**Conclusion:**

Persistent sex disparities in HF mortality underscore the need for gender-sensitive approaches to cardiovascular disease prevention, diagnosis, and treatment. Public health policies must prioritize HF and integrate sex-specific considerations to address inequities and improve outcomes for both women and men.

## Introduction

Cardiovascular diseases remain the leading cause of death in many countries, with heart failure (HF) playing a significant role. According to European and American clinical guidelines, HF is defined as a clinical syndrome characterized by signs, such as pulmonary crackles, elevated jugular venous pressure, and peripheral edema, as well as symptoms, such as dyspnea and fatigue, resulting from reduced cardiac output or increased intracardiac pressures. These changes stem from structural or functional alterations of the cardiovascular system (1). HF encompasses a wide spectrum of patients, who are primarily classified based on left ventricular ejection fraction (LVEF). Currently, HF represents a major public health concern due to its high incidence and prevalence, which contribute to substantial hospitalization and mortality rate (2). The global prevalence of HF has doubled over the past two decades, with a particularly rapid rise in low and middle income countries (3). This increase is attributed to several factors (4). First, HF is more common in older adults, a demographic group that is growing worldwide. Second, improvements in the treatment of acute myocardial infarction and hypertension—the leading causes of HF—have increased patient survival. Finally, evidence indicates that current therapies, such as sacubitril-valsartan and sodium-glucose cotransporter-2 (SGLT2) inhibitors, significantly improve survival in HF patients (5).

Sex plays a critical role in the development and clinical course of HF (6, 7). Women are more likely to present with HF with preserved ejection fraction (HFpEF), whereas men more frequently develop HF with reduced ejection fraction (HFrEF). Risk factors such as obesity, diabetes, psychological stress, lower socioeconomic status, and lower educational attainment are more prevalent in women. Additionally, women may experience sex-specific conditions such as peripartum cardiomyopathy and cardiotoxicity from chemotherapy for breast cancer. It is important to clarify that, although HFpEF is the predominant HF phenotype in women, the cardiotoxic effects of radiation and chemotherapy—particularly in breast cancer treatment—are more commonly linked to the development of HFrEF rather than HFpEF.

Despite these differences, clinical trials that inform current HF management guidelines have historically enrolled more men, limiting the generalizability of findings to women (8). Overall, women—regardless of country, geographic region, or ejection fraction—report more severe symptoms and poorer quality of life compared to men. However, hospitalization rates are similar between sexes, and women tend to have lower mortality rates. The reasons for this disparity remain unclear.

In Spain, HF is consistently the third leading cause of cardiovascular death, following coronary artery disease and stroke. In 2023, 18,954 people died from HF (7,721 men and 11,233 women), representing 4.4% of all deaths and 16.6% of cardiovascular deaths (9). The estimated prevalence of HF in Spain is 2.3%, affecting 4.7 to 6.8% of individuals over the age of 45 and 16% of those over 75 (9–11). HF is the leading cause of hospitalization among individuals over 65 years old, accounting for more than 25% of admissions due to cardiac disease. Furthermore, approximately 10% of patients hospitalized for HF die before discharge. One-year mortality after diagnosis is around 20%, increasing to 50% within 5 years. In Spain, HFpEF is the most prevalent phenotype in primary care, although diagnosis and treatment initiation are more frequently performed in hospital settings (12, 13).

Due to the differences in heart failure (HF) between women and men in terms of epidemiology, pathogenesis, response to treatment, and quality of care, this study analyzes HF mortality data according to sex (8, 14). In 2000, in Spain, HF accounted for 4% of all deaths and 10% of cardiovascular deaths in men. In women, these figures were 8 and 18%, respectively. In 2023, HF accounted for 3.5% of all deaths and 13.9% of cardiovascular deaths in men and 5.2 and 18.6% in women, respectively (9).

This study describes trends in HF mortality among men and women in Spain over the first 24 years of the 21st century, with a detailed analysis of sex differences across five-year age groups.

## Materials and methods

### Study design and data sources

This study is a descriptive time series analysis of annual heart failure (HF) mortality in Spain among adults aged 50 years and older, from 2000 to 2023. Data are disaggregated by sex and five-year age groups. Annual mortality data were obtained from the Statistics on Deaths According to Cause of Death provided by the Spanish National Institute of Statistics (INE). The INE reports mortality based on the underlying cause of death, in accordance with the World Health Organization’s International Classification of Diseases (ICD). Data are collected through three official instruments: (1) the Medical Death Certificate/Statistical Death Bulletin, (2) the Judicial Death Statistical Bulletin, and (3) the Birth Statistical Bulletin.

### Variables

The primary variables analyzed were:

- Annual number of deaths due to HF, disaggregated by sex and five-year age groups.
- Crude and age-adjusted HF mortality rates by sex and five-year age groups.

### Data collection

A dataset was constructed using mortality data from the INE’s Statistics on Deaths by Cause of Death. HF-related deaths were identified using ICD-10 codes I50–I50.9 for both sexes. The study period spans 24 years, from 2000 to 2023 (the most recent year available). Age-adjusted mortality rates were calculated for individuals aged 50 years and older, using five-year age strata.

### Statistical analysis

The objective of the trend analysis was to evaluate the overall trajectory of HF mortality over time, including detection of linear trends, seasonality, cyclical patterns, and irregular fluctuations.

- Descriptive analysis: Absolute and relative frequencies were calculated for categorical variables, and measures of central tendency and dispersion were calculated for continuous variables.
- Group comparisons: Differences between men and women were assessed using Pearson’s correlation coefficient, Student’s t-test, and chi-square tests, as appropriate.
- Time series analysis: An ARIMA (1, 0, 1) model was applied to model the temporal evolution of HF mortality and to identify underlying trends and autocorrelation structures in the data.

### Ethical considerations

Because the data are anonymized, aggregated, and publicly available, approval by an ethics committee and informed consent were not required.

## Results

**Figure 1:**
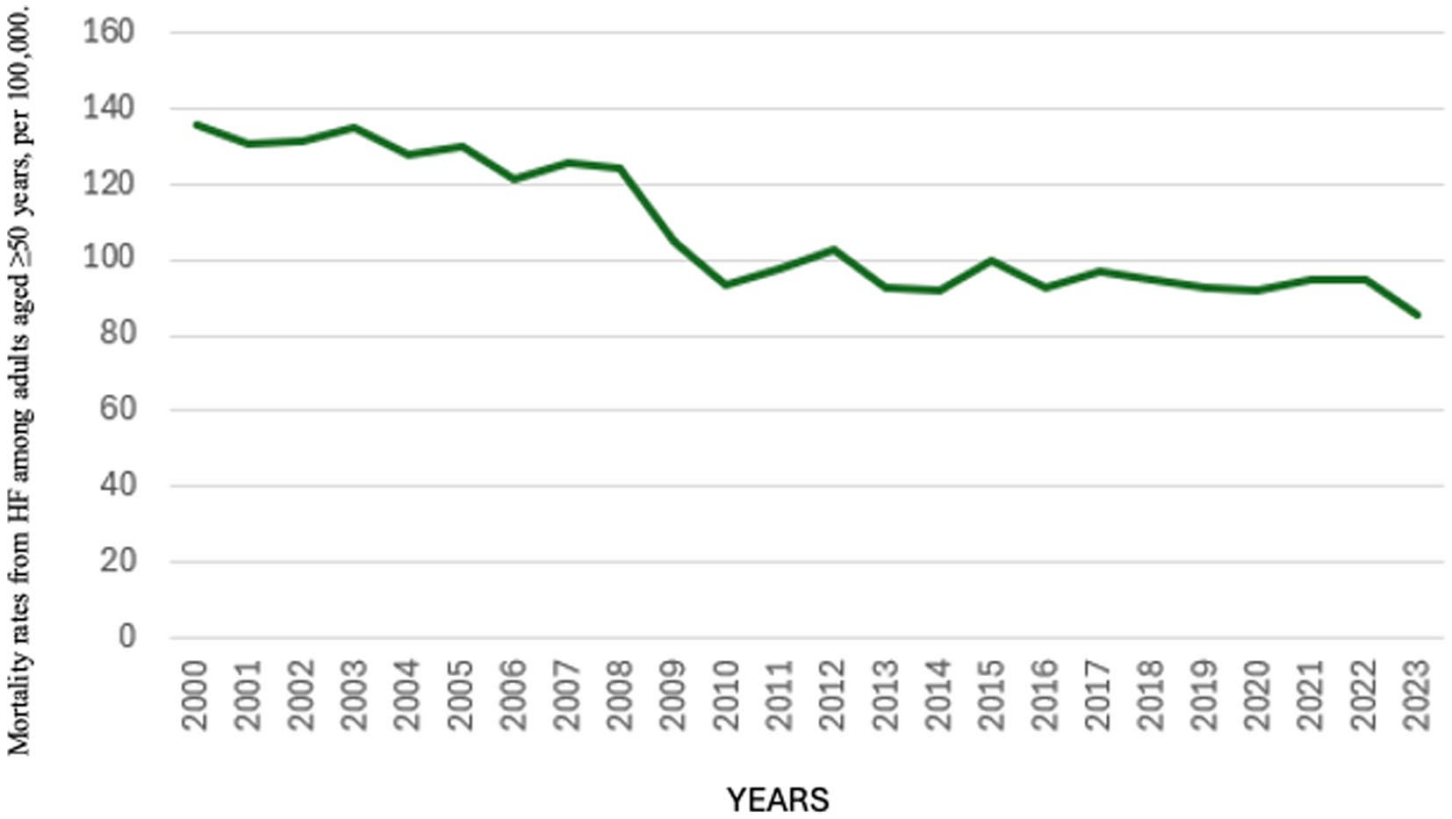
Age-adjusted mortality rates from heart failure among adults aged ≥50 years, per 100,000 population; Spain, 2000–2023. This figure displays the overall trend in age-adjusted mortality rates from heart failure in the population aged 50 years and older. The data reflect the national-level burden and its evolution over time, providing the epidemiologic context for subsequent sex- and age-specific analyses.

Age-adjusted mortality rates due to heart failure (HF) among individuals aged 50 years and older in Spain showed a slight overall decline over the 24-year study period, with a more pronounced reduction between 2000 and 2008, followed by a more moderate decrease from 2009 to 2023 (Figure 1).

Between 2000 and 2008, adjusted HF mortality rates ranged from 121 to 135 deaths per 100,000 population. From 2009 to 2023, the rates declined further, ranging from 85 to 102 deaths per 100,000. The highest rate was recorded in 2000 (135.94 per 100,000), and the lowest in 2023 (85.37 per 100,000) (Figure 1).

**Figure 2:**
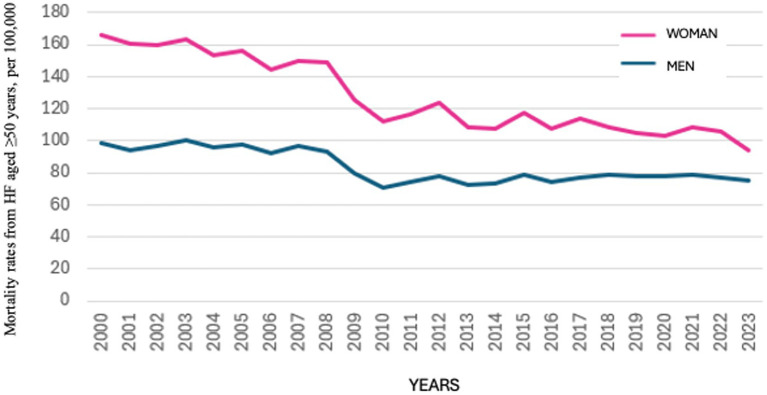
Comparative age-adjusted mortality rates from heart failure in women and men aged ≥50 years, per 100,000 population; Spain, 2000–2023. This figure presents sex-disaggregated age-adjusted mortality rates from heart failure among adults aged 50 years and older. Separate curves for women (pink line) and men (blue line) allow for the assessment of persistent or changing mortality differentials between sexes over the study period.

When disaggregated by sex, HF mortality in women declined from 165.72 per 100,000 in 2000 to 93.99 per 100,000 in 2023. Among men, the corresponding rates were 98.89 per 100,000 in 2000 and 75.09 per 100,000 in 2023 (Figure 2).

**Figure 3:**
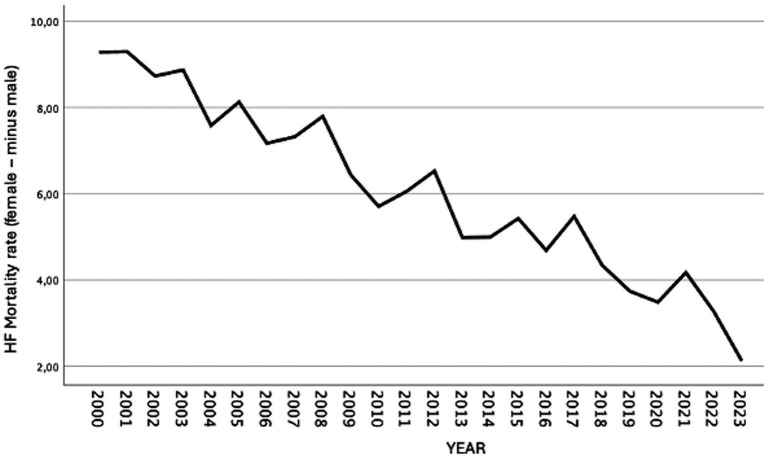
Temporal evolution of the sex disparity in heart failure mortality (female minus male rate) among adults aged ≥50 years; Spain, 2000–2023. The sex disparity was calculated by subtracting the age-adjusted mortality rate in men from that in women for each calendar year. Positive values indicate higher mortality rates in women, while negative values indicate higher mortality rates in men. Trends are shown to highlight changes in the magnitude and direction of the sex gap over time.

Throughout the study period, the difference in HF mortality rates between women and men gradually narrowed (Figure 2). The gender gap—defined as the difference in HF mortality between women and men aged 50 years and older—decreased consistently over time (Figure 3). The mean HF mortality rate among women during the study period was 127.53 per 100,000 population, which was 34.4% higher than the mean rate among men (83.66 per 100,000). However, this gap declined from a 40% difference in 2000 to 20.1% in 2023. Despite the reduction, HF mortality remained consistently higher in women throughout the entire period.

Age-adjusted HF mortality rates by five-year age groups in adults over 50 years of age in Spain (2000–2023) are presented as pooled data and disaggregated by sex.

As expected, HF mortality increased progressively with age. Table 1 presents the mean adjusted mortality rates for each five-year age group, calculated over the 24-year study period, for both men and women aged 50 years and older. The overall mean mortality rate was 540.5 per 100,000 in men and 601.3 per 100,000 in women. The difference between sexes was statistically significant (*p* < 0.0001).

**Table 1:** Mean age-adjusted heart failure mortality rates (per 10,000 population) by sex and five-year age group among adults aged ≥50 years; Spain, 2000–2023.

Age group (years)	Women	Men	Female-to-male ratio
50–54	2.2	7.7	0.29
55–59	3.6	10.7	0.34
60–64	6.3	15.6	0.40
65–69	12.7	25.3	0.50
70–74	32.2	48.7	0.66
75–79	88.4	105.9	0.83
80–84	270.9	268.3	1.01
85–89	723.5	646.7	1.12
90–94	1661.6	1485.7	1.12
≥95	3212.1	2790.9	1.15

Values represent the mean of annual age-adjusted mortality rates from 2000 to 2023 in each five-year age group, expressed per 10,000 population. Rates are disaggregated by sex. All data were obtained from the Spanish National Statistics Institute (Instituto Nacional de Estadística, INE).

Mortality due to HF was consistently higher in men in the age groups between 50 and 79 years. However, from the age of 80 onward, mortality rates between men and women converged. In the oldest age groups, mortality was higher in women.

**Figure 4:**
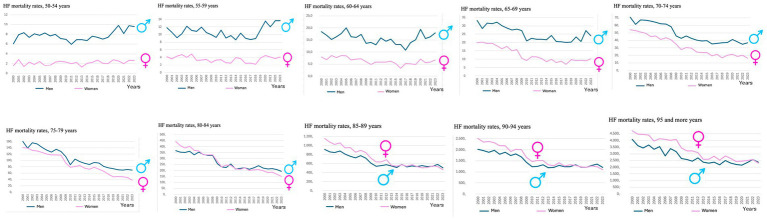
Trends in age-adjusted heart failure mortality rates by sex and five-year age groups among adults aged ≥50 years, highlighting differences in female and male patterns; Spain, 2000–2023. The figure includes ten panels, each representing a five-year age group (from 50 to 54 to ≥95 years). Within each panel, the time trend of age-adjusted mortality rates is shown separately for women (pink line) and men (blue line). This visualization allows for a comparative analysis of sex-specific mortality trajectories across age strata over the study period.

**Figure 5:**
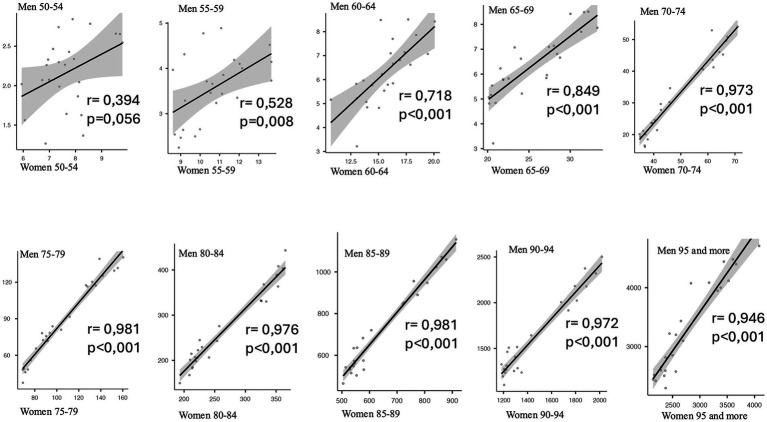
Correlation between female and male age-adjusted heart failure mortality rates across five-year age groups (50–54 to ≥95 years), with Pearson’s r and *p*-values; Spain, 2000–2023. Each subplot represents one five-year age group and displays the linear correlation between the age-adjusted mortality rates for women and men across the study period. Pearson’s correlation coefficient (r) and corresponding *p*-value are shown in each panel. This analysis aims to evaluate the strength and consistency of the association in sex-specific mortality patterns across age.

Figures 4, 5 illustrate the temporal evolution of HF mortality by sex and five-year age group from 2000 to 2023. As shown in Figure 4, mortality was higher in men than in women in the 50–79 age groups, although the gap progressively narrowed with advancing age. In the 80–84 age group, rates were similar between sexes, and in the older age groups, women exhibited higher mortality than men.

Figure 5 provides a graphical and analytical depiction of the relationship between male and female HF mortality across five-year age groups above age 50, using scatter plots, adjusted linear regression lines, and Pearson correlation coefficients, along with their statistical significance. The results show a progressive increase in the correlation coefficients across age groups, indicating an improved linear fit and a consistent relationship in the evolution of mortality patterns between both sexes, with high levels of statistical significance from the age of 60 onwards (r ranging from 0.72 to 0.97 and a *p* value *p* < 0.001 in all these age subgroups).

## Discussion

The global burden of cardiovascular disease (CVD) continues to rise (15), largely driven by population aging. Nonetheless, the decline in age-standardized cardiovascular mortality rates reflects substantial advances in medical care and secondary prevention strategies following diagnosis. In our study, overall heart failure (HF) mortality in Spain declined over recent decades, with a marked reduction observed between 2000 and 2008, followed by a more gradual decrease in subsequent years. Despite this overall improvement, women exhibited a 34.4% higher average HF mortality rate compared to men; however, this gender gap narrowed significantly over the study period. Age-stratified analyses revealed that men aged 50 to 79 had higher HF mortality, whereas women aged 80 and older experienced greater mortality rates. These findings from our analysis highlight important sex- and age-related disparities in HF outcomes that warrant further investigation.

### HF mortality trends in men and women

In 2021, age-standardized mortality rates for diseases of the circulatory system were higher in men than in women across all European Union countries. However, specific differences exist among the various conditions grouped under cardiovascular diseases (16). Most epidemiological studies have reported declining trends in HF mortality in recent decades. For example, between the periods 1959–1969 and 1990–1999, HF mortality in Europe decreased from 70 to 59% in men and from 57 to 45% in women (17).

The downward trend in HF mortality rates observed among men and women in this study, spanning the early 21st century in Spain, is consistent with trends seen in most European countries. This contrasts with data from the United States, where HF mortality has been rising since 2012, with a particularly marked increase during 2020–2021 (18). In fact, by 2021, age-adjusted HF mortality rates in the U.S. exceeded those recorded in 1999 and accounted for 45% of all cardiovascular deaths. In contrast to our findings in Spain—where mortality has been persistently higher in women—U.S. data from 2022 showed lower HF mortality in women (72.8 per 100,000 population) than in men (126.9 per 100,000 population) (18).

Similarly, in France in 2022, there were 24,645 deaths with HF listed as the primary cause, with higher age-standardized mortality rates in men (41.8 per 100,000) than in women (32.9 per 100,000) (19). In Sweden, Eriksson et al. (2024) reported significantly higher HF mortality rates in men than in women between 2000 and 2014 (*p* < 0.0001) (20). Likewise, in the Netherlands, data from 2017 showed higher HF mortality among men, although the prevalence was greater in women (21).

In contrast to the trends observed in the United States, France, Sweden, and the Netherlands, HF-adjusted mortality rates in Spain have consistently been higher in women throughout the study period. In 2023—the most recent year with available data—the mortality rate was 93.99 per 100,000 in women and 75.09 per 100,000 in men.

On the other hand, some studies have reported no significant sex-based differences in HF mortality. For instance, Averbuch et al. (22) analyzed HF mortality among hospitalized patients in Canada and found no differences in mortality, readmission, or emergency department visits between men and women. However, they did observe notable differences in clinical management: despite having comparable clinical profiles, men were more likely to receive specialized care and undergo surgical procedures, whereas women were more often managed conservatively with supportive treatment (22).

### HF differences in men and women

Several studies suggest that the natural history of heart failure (HF) differs between men and women, but the interplay between biological sex, sociocultural gender determinants, and the clinical manifestations and outcomes of HF remains incompletely understood (6, 16, 23, 24). Beyond genetic and hormonal differences, social roles—including occupational activities, exposure to risk factors, and physical activity levels—may influence disease susceptibility, healthcare-seeking behavior, access to services, and utilization of the healthcare system.

In this context, a recent meta-analysis by Qiu et al. (7) reported that women with HF had a lower risk of all-cause mortality (HR: 0.83), cardiovascular mortality (HR: 0.84), and HF-related hospitalization (HR: 0.94) compared to men. Similarly, Prasad et al. (3) found that although women with HF experienced more severe symptoms and lower quality of life, their hospitalization rates were comparable to those of men, and their adjusted mortality was significantly lower. These findings were consistent across countries, economic contexts, geographic regions, and ejection fraction subtypes, suggesting that the survival advantage in women remains only partially explained.

Rodríguez-Artalejo et al. (4) noted that sex-related differences in HF survival tend to diminish after adjusting for ejection fraction and blood pressure. Compared to men, women with HF tend to be older, more symptomatic, and more frequently hypertensive or diabetic, but less often diagnosed with ischemic heart disease (4).

In a large cohort from the Swedish HF Registry, Stolfo et al. (25) found that women were more likely to have HF with preserved ejection fraction (HFpEF), were older, and had a higher prevalence of hypertension and chronic kidney disease. They were less likely, however, to have diabetes or ischemic heart disease. After multivariable adjustment, women had a significantly lower risk of adverse events than men, regardless of ejection fraction. The higher symptom burden in women may be partly explained by delayed diagnosis or greater exposure to psychosocial stressors.

Unlike heart failure with reduced ejection fraction (HFrEF), therapeutic evidence for improving survival in HFpEF remains limited. Until recently, effective disease-modifying therapies were lacking. However, emerging data support the role of sodium-glucose cotransporter 2 (SGLT2) inhibitors and finerenone in reducing cardiovascular events and mortality in this population (26–29).

Additionally, Regitz-Zagrosek and Gebhard (6) reported that women are more susceptible to adverse effects from cardiovascular drugs than men. Differences in pharmacodynamics and pharmacokinetics—possibly driven by sex hormones, age, comorbidities, genetic polymorphisms, and polypharmacy—may necessitate sex-specific dosage adjustments or treatment strategies for optimal care (6).

The age-related difference in HF mortality between sexes observed in this Spanish study is consistent with findings from Japan (30), where the mean age at HF-related death was 74.0 ± 12.8 years for men and 80.8 ± 11.3 years for women.

In addition to cross-country variability, diagnostic delays may also be influenced by sex-specific factors. Women, who are more frequently affected by heart failure with preserved ejection fraction (HFpEF), often present with nonspecific symptoms and multiple comorbidities that complicate early recognition. Several studies have shown that women with HF are more likely to experience delayed diagnosis, receive fewer diagnostic tests such as echocardiograms, and have lower adherence to guideline-recommended treatments compared to men (31, 32). These disparities may reflect underlying biases in clinical assessment and contribute to poorer outcomes. Although our study does not include clinical management data, these sex-related factors should be taken into account when interpreting the observed differences in HF mortality.

Spain ranks among the countries with the highest life expectancy in Europe. In 2023, the average life expectancy was 84 years: 81.1 years for men and 86.3 years for women (9). The gender gap in life expectancy, historically favoring women, has progressively narrowed in the past two decades—from 6.8 years in 2002 to 5.4 years in 2022, mirroring the EU-27 average (9). The higher HF mortality observed in Spanish women may thus be partially attributable to the overrepresentation of women in the oldest age groups, where mortality is naturally higher, and to Spain’s relatively high longevity compared to other European countries.

### Limitations

As with other diseases and causes of death, knowledge of the epidemiology of heart failure (HF) depends on the quality of diagnostic criteria and their accurate documentation. Data obtained from the National Institute of Statistics (INE) cause of death statistics depend on correctly identifying and coding the primary cause of death according to the International Classification of Diseases (ICD) criteria. Consequently, fatalities where HF is not documented as the primary cause are excluded from statistics that record the primary cause. The mortality coding rules may prioritize assigning the cause of death to ischemic heart disease rather than HF, which could lead to an underestimation of the magnitude of HF mortality in both sexes (4).

Additionally, although the study identifies a sex gap in HF mortality—largely explained by differences in age and HF phenotype—this should be interpreted in light of the distinct clinical profile of heart failure with preserved ejection fraction (HFpEF), which disproportionately affects women. HFpEF is often characterized by a slower and more insidious progression, frequently resulting from cumulative exposure to cardiovascular risk factors, and is commonly subject to delayed diagnosis. Moreover, while the limited availability of effective treatments for HFpEF is acknowledged, the study design does not provide information on patients’ pharmacological management or adherence to guideline-recommended therapies. These limitations may have influenced the observed sex-specific patterns and should be considered when interpreting the results.

## Conclusion

From 2000 to 2023, Spain’s age-adjusted heart failure mortality rates were higher for women than for men. This phenomenon stands in contrast to the trend observed in the United States and most European countries, where mortality rates are higher for men.

The magnitude of heart failure mortality and the differences between sexes underscore the need for more personalized care, as well as the importance of considering it in the cardiovascular health strategies of the national health system. This can be achieved by coordinating the different levels of care to make it easier for specialists and primary care physicians to provide differentiated care and manage chronic patients.

The elevated mortality rate from heart failure, in conjunction with the observed disparities between sexes, underscores the necessity for a more customized healthcare approach, gender-specific, and the integration of heart failure management within the broader cardiovascular health strategies of national health systems. This objective can be accomplished by orchestrating the various levels of care to facilitate the provision of specialized care and the management of chronic patients by primary care physicians.

## Data Availability

The original contributions presented in the study are included in the article/supplementary material, further inquiries can be directed to the corresponding author.
